# Crystallization of ZnO thin films via thermal dissipation annealing method for high-performance UV photodetector with ultrahigh response speed

**DOI:** 10.1038/s41598-020-79849-z

**Published:** 2021-01-11

**Authors:** Dongwan Kim, Jae-Young Leem

**Affiliations:** Department of Nanoscience & Engineering, Inje University, 197, Inje-ro, Gimhae-si, 621-749 Gyeongsangnam-do Republic of Korea

**Keywords:** Materials science, Materials for devices, Structural materials

## Abstract

ZnO-based ultraviolet (UV) photodetector can be easily fabricated by using sol–gel spin-coating method, however, the crystallization of amorphous state ZnO thin films is necessary to fabricate high performance UV photodetector. Thus, we devised a thermal dissipation annealing (TDA) method in which the heat transfer to the ZnO thin films can be synchronized with the heat release from the substrate. It was found that sol–gel spin-coated ZnO thin films can be crystallized through the mobility difference of ZnO molecules placed at the surface of ZnO thin films. Also, UV photodetector based on ZnO thin films annealed with the TDA method exhibited faster rise and decay time constant (τ_r_ = 35 ms and τ_d_ = 73 ms, respectively), a higher on/off current ratio, and reproducible photocurrent characteristics compared to that of the ZnO thin films annealed by using furnace and IR lamp. Therefore, these results indicated that the TDA method is a feasible alternative route for the fabrication of ZnO based high performance optoelectronic devices.

## Introduction

Recently, ultraviolet (UV) photodetectors have attracted considerable research interest in the last few years because of their great potential and commercial impact in a wide variety of fields, such as secure space-to-space communications, pollution monitoring, water sterilization, flame sensors, and missile plume detection applications^1,2^. Various semiconductors, such as Si, ZnS, and ZnO, have been used for UV photodetectors. Among them, Si-based UV photodetectors exhibit a fast response; however, the narrow band gap of Si deteriorates the sensitivity and selectivity of low energy photons (visible and infrared (IR) light)^3^. Therefore, when Si-based UV photodetectors are used, a complex filter is required to avoid noise related to low electron energies. Additionally, for the accurate detection of Si-based UV photodetectors, an ultrahigh vacuum and high voltage are required^4^. In the case of ZnS-based UV photodetectors, a complex filter is not required due to the wide band gap of ZnS (3.91 eV), but ZnS-based UV photodetectors show weak value and poor stability of photocurrent, and they only react to UV light with wavelengths shorter than 335 nm^5^. In contrast, ZnO-based UV photodetectors are free from the requirement of complex filter due to their direct wide band gap of 3.37 eV and large exciton binding energy of 60 meV^6–8^. Over the years, various growth method such as sol–gel spin-coating method, metal–organic chemical vapor deposition, molecular beam epitaxy (MBE), pulsed laser deposition, electrodeposition, and hydrothermal method have been used to fabricate the ZnO based UV photodetector. Among them, the deposition of ZnO thin films by using the sol–gel spin-coating method has a variety of advantages, such as simplicity, easy control of the doping level, straightforward solution concentrations, the production of homogeneous films, and feasibility of large-area deposition without the use of expensive and complicated equipment that is required with other methods^9–12^. However, despite of these advantages, the deposition of ZnO thin films by using sol–gel spin-coating method has a critical drawback: crystallization of amorphous state ZnO thin films. When the ZnO thin films are deposited on substrate by using sol–gel spin-coating method, the crystal state of ZnO thin films is amorphous state which has poor crystallinity and defect sites bound the free electron^13^. Thus, many researchers have used the various annealing method to transform the amorphous state of ZnO thin films into crystalline state. The most common annealing method for crystallization of sol–gel spin-coated ZnO thin films is thermal annealing in the furnace. But, the thermal annealing through furnace is progressed in high temperature (above 500 °C) and the lattice misfits is generated due to the difference of coefficient of thermal expansion between ZnO thin films and substrate^14,15^. Kang and Jo et al. deposited the ZnO thin films by using sol–gel spin-coating method and successfully crystallized the ZnO thin films by using laser annealing method^16,17^. But, when the sol–gel spin-coated ZnO thin films are annealed by using laser annealing, although the crystallinity of ZnO thin films are improved compared to the ZnO thin films annealed by using furnace, the transparency of ZnO thin films decreased because the increase of grain boundary density causes the increase of scattering photons. In addition, before the laser annealing method, the rapid thermal annealing process is required to crystallize the amorphous state ZnO thin films completely and this additional annealing process increases the number of oxygen vacancies which deteriorates the performance of ZnO based optoelectronics. Therefore, to fabrication of high-performance UV photodetector, a novel annealing method for crystallization of sol–gel spin-coated ZnO thin films, which can crystallize completely the amorphous state of ZnO thin films with low density of defects, must be developed.

In this study, we developed a novel annealing method, the thermal dissipation annealing (TDA) method, that eliminates the thermal energy from substrates through a thermal dissipation annealing system combined with an IR lamp and cold plate to restrict the defect formation in the ZnO lattice through mobility difference of ZnO molecules. Furthermore, to confirm the annealing efficiency of the TDA method, we deposited the ZnO thin films by using the sol–gel spin-coating method and annealed the ZnO thin films at 500° by using furnace, IR lamp, and TDA methods. In addition, we fabricated ZnO-based UV photodetectors to suggest an effective route for the fabrication of ZnO based high-performance UV photodetectors.

## Results and discussion

### Annealing process and mechanism of the TDA method

**Figure 1:**
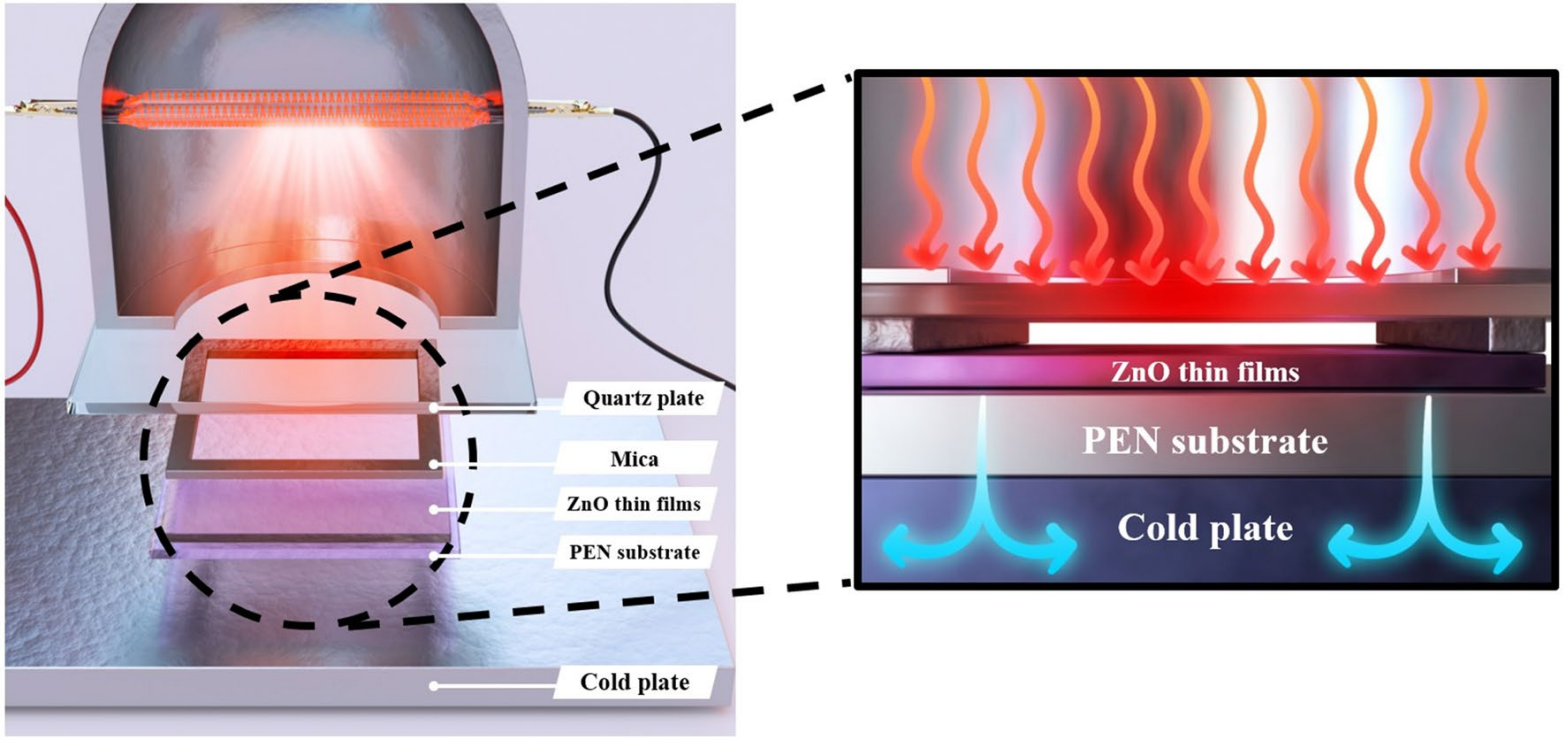
Schematics images of the thermal dissipation annealing (TDA) systems for annealing ZnO thin films deposited onto PEN substrate (figures not drawn to scale). The schematic images were prepared by first author Dongwan Kim.

**Figure 2:**
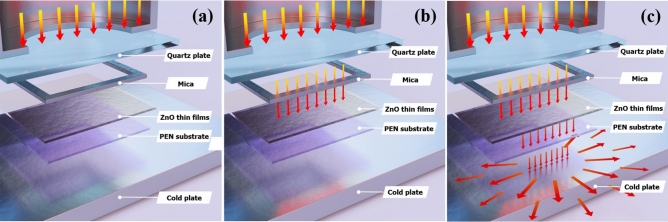
Schematics images for annealing process of TDA method composed of three steps: **(a)** generation of thermal energy, **(b)** flow of thermal energy, and **(c)** dissipation of thermal energy. The schematic images were prepared by first author Dongwan Kim.

Figure 1 shows a schematic illustration of TDA method for the crystallization of amorphous ZnO thin films for restriction of the defects sites formation. The annealing process with the TDA method is as follows. First, before starting the annealing process, the temperature of the cold plate is set to − 10 °C. When the temperature of the cold plate reaches − 10 °C, the ZnO thin films deposited onto a Si substrate by using the sol–gel spin-coating method is placed on the cold plate to decrease the temperature of the Si substrate. After the ZnO thin films are placed on a cold plate for 30 s, a thin mica plate with a hollow square at its center is placed on the ZnO thin films, and a quartz plate is placed on the mica plate to form a separate space on the surface of the ZnO thin films. The formation of a separate space through the mica plate and quartz plate by placing them on the ZnO thin films, as well as a cooling time of 30 s, are essential aspects of the TDA method. The separate space on the ZnO thin films surface is essential to prevent the formation of moisture onto the ZnO thin films surface. In other words, when the ZnO thin films are annealed by using the TDA method without a separate space, a temperature below the dew point on the ZnO thin films surface for a cooling time of 30 s allows moisture to form and the generated moisture decreases the annealing efficiency and heat transfer rate of the TDA method. The optimum TDA cooling time (30 s) also facilitates the annealing of ZnO thin films with mobility difference of ZnO molecules. If the cooling time is shorter than 30 s, the mobility difference between ZnO molecules at the surface and bottom of ZnO thin films is rarely occurred and the defects sites which deteriorates the performance of ZnO-based optoelectronics is generated due to the rapid rate of the temperature increase. In contrast, if the cooling time is longer than 30 s, the surface of the ZnO thin films freezes and interrupts the crystallization of the amorphous ZnO thin films. Therefore, to crystallize amorphous ZnO thin films by using the TDA method, a suitable cooling time and isolated space on the ZnO thin films surface is essential. Furthermore, to confirm the annealing mechanism of the TDA method, we categorized the TDA process into three steps: (i) the generation of thermal energy (Fig. 2a), (ii) the flow and absorption of thermal energy (Fig. 2b), and iii) the dissipation of thermal energy (Fig. 2c). In the first step (the generation of thermal energy), the thermal energy is generated from an IR lamp that is placed above the quartz plate. Second, the generated thermal energy passes through the quartz and mica plates, and this thermal energy reaches the surface of ZnO thin films. At this point, some of the thermal energy is absorbed into the surface of ZnO thin films and used to crystallize the amorphous ZnO thin films, whereas the thermal energy unabsorbed by the ZnO thin films passes through it and reaches the substrate. However, this thermal energy is directly dissipated through the cold plate, and this thermal dissipation enables annealing of ZnO thin films at high temperatures above 500 °C. As a result, these three steps (the generation of thermal energy, the flow and absorption of thermal energy, and the dissipation of thermal energy) can crystallize amorphous ZnO thin films at high temperatures and prevent the formation of defect sites.

### Morphological, structural, and PL analysis of ZnO thin films

**Figure 3:**
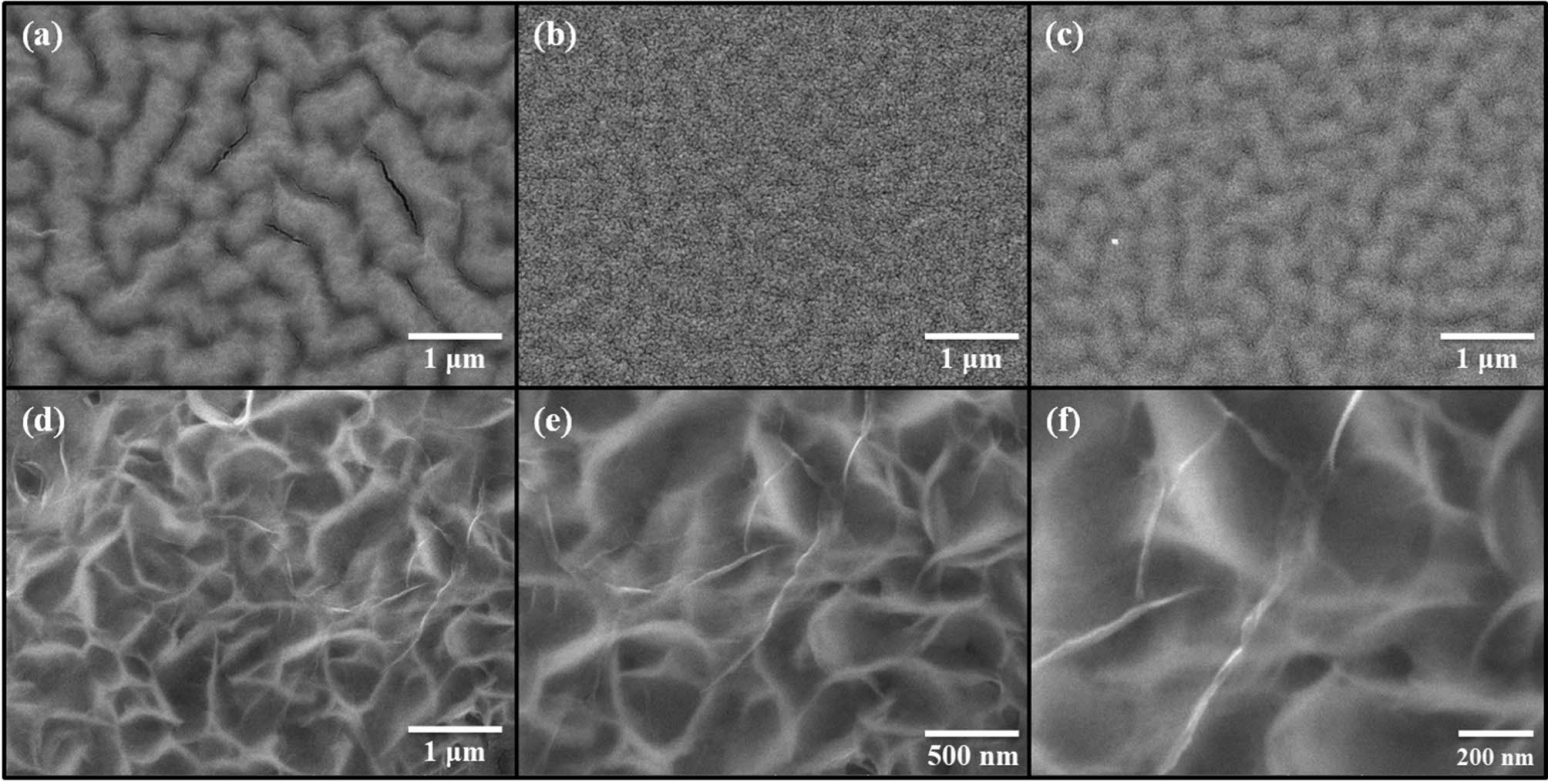
FE-SEM images of sol–gel spin-coated ZnO thin films annealed by using various method. **(a)** Non-annealed ZnO thin films, **(b)** furnace, **(c)** IR lamp, and **(d–f)** TDA method.

**Figure 4:**
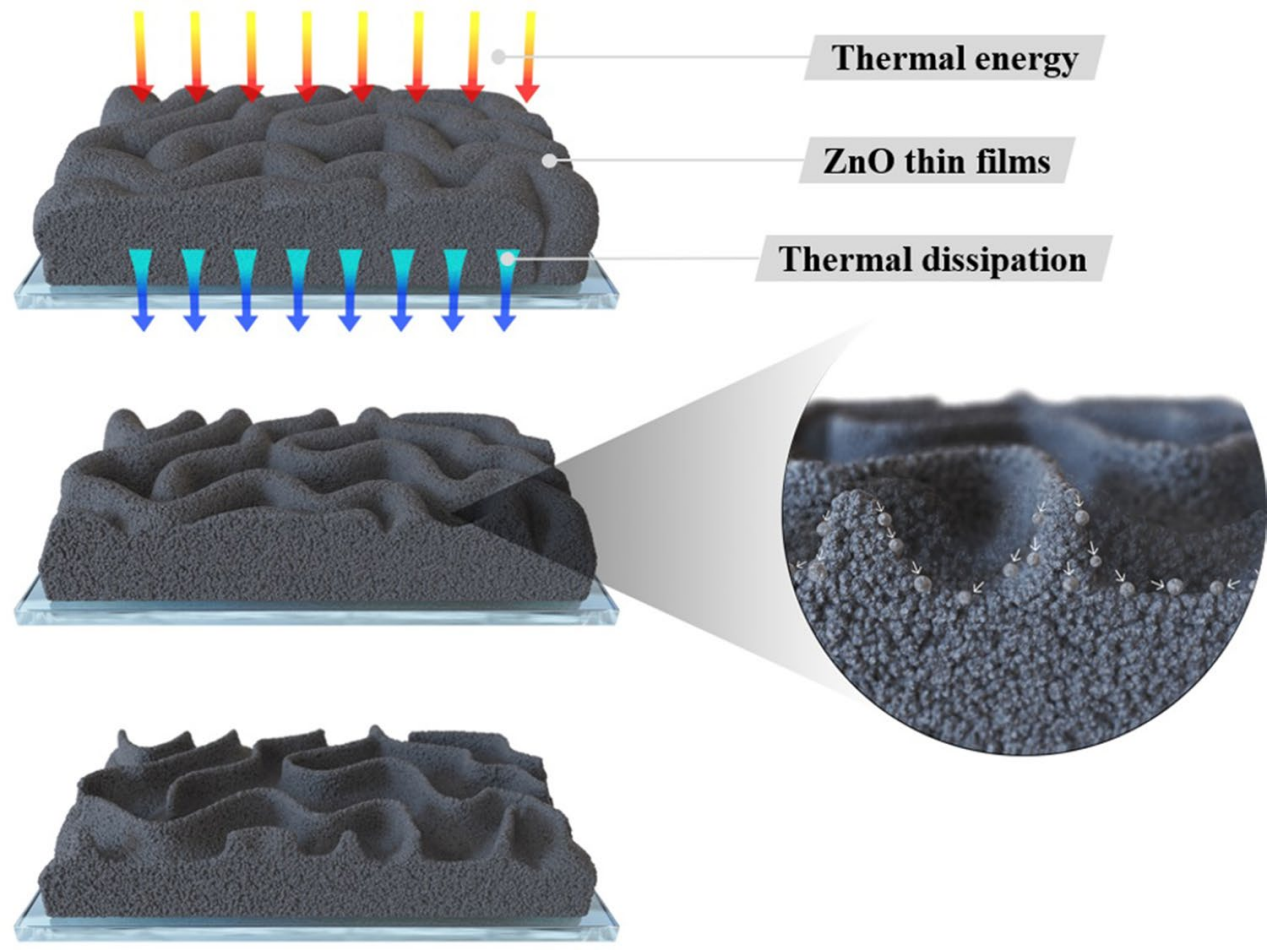
Schematics images of ZnO molecules movement during TDA annealing process. The schematic images were prepared by first author Dongwan Kim.

**Figure 5:**
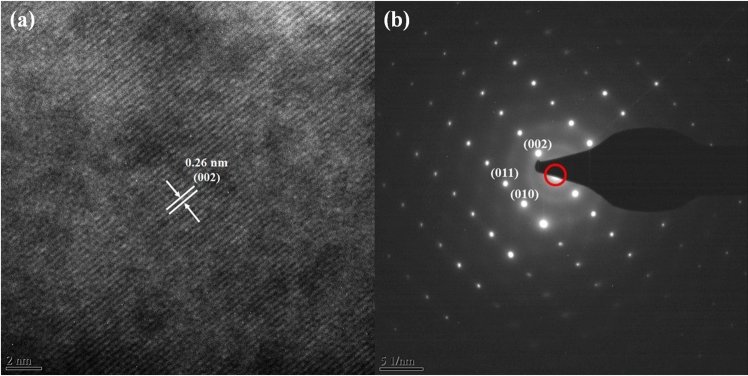
**(a)** FE-TEM images and **(b)** SAED patterns of sol–gel spin-coated ZnO thin films annealed by using TDA.

Figure 3 shows the field-emission scanning electron microscopy (FE-SEM) images of the unannealed ZnO thin films and annealed ZnO thin films that used the furnace, IR lamp, and TDA method. As shown in Fig. 3, the unannealed ZnO thin films were found to have a wrinkled network structure consisting of dense grains from agglomerated ZnO nanoparticles and some cracks were observed between wrinkled network structures. With respect to ZnO thin films annealed at 500 °C by using furnace and IR lamp, the wrinkled network structure was also observed, but the nanoparticle size increased, and the cracks disappeared, as confirmed in Fig. S1. In general, sol–gel spin-coated ZnO thin films are amorphous before annealing and when the ZnO thin films are annealed at high temperatures (above 500 °C), crystallization progresses. During the annealing process, the nanoparticle size in the ZnO thin films increased due to Ostwald ripening, and an increase in the nanoparticle size and disappearance of cracks in the ZnO thin films annealed in the furnace and by the IR lamp proved the crystallization of the ZnO thin films^18^. The X-ray diffraction (XRD) patterns of the ZnO thin films in Fig. S2 proved that the non-annealed ZnO thin films were amorphous, and after annealing by using furnace and IR lamp, the crystal state of the ZnO thin films transformed from amorphous to polycrystalline. Interestingly, in the case of the ZnO thin films annealed by using the TDA method, graphene-like nanosheets were observed. Herein, the important factor for the graphene-like nanosheet formation with the TDA method may be the cooling time before the annealing process. In general, the surface molecules of thin films deposited onto a low-temperature substrate exhibit a short migration length due to the low temperature of the substrate, whereas when thin films are deposited on a high-temperature substrate, the surface molecules of the thin films exhibit enhanced diffusion^19^. Chaâbane et al. reported that a low substrate temperature increases the density of thin films composed of nanocrystals due to an increase in the residence time^20^. Similarly, during the TDA process, the sol–gel spin-coated ZnO thin films were cooled for 30 s before the annealing process. There is a temperature difference between the surface and bottom of the ZnO thin films that changed the mobility of the ZnO molecules at the surface and bottom of the wrinkled network structures. As the annealing proceeded, the temperature difference increased and the difference in the mobilities of the ZnO molecules also incraeased. As a result, the ZnO molecules at the surface of the wrinkled network structures had an elevated mobility owing to the large amount of thermal energy and high temperature and moved to the bottom of the wrinkled network structures to reach a thermal equilibrium state. On the other hand, the ZnO molecules at the bottom of the wrinkled network structures, which had a decreased mobility owing to a small amount of thermal energy and low temperature, did not move to the surface of the wrinkled network structures. As the annealing continued, additional ZnO molecules at the surface moved to the bottom of the wrinkled network structures, and the thickness at the edge of the wrinkle network structures became thinner than that of the center of the wrinkled network structures due to the increased amount of ZnO molecule movement, as shown in Fig. 4. At the end of the annealing process, the thermal energy required for crystallization was transferred to the ZnO molecules placed at the bottom of the wrinkled network structures through the relatively thin wrinkled network structure at the edge, which caused the crystallization of the ZnO thin films at the bottom of the wrinkled network structures. However, in the case of the ZnO thin films annealed by using the furnace and IR lamp, the nanoparticle size increased, but a morphology change due to the movement of the ZnO molecules was not observed. In other words, when the ZnO thin films were annealed without a cold plate, the nanoparticle size of ZnO thin films increased because of Ostwald ripening, and the cracks between the wrinkled network structures decreased; however, there was no change in the morphology because the mobility of the ZnO molecules at the surface and bottom of wrinkled network structures did not differ. Therefore, the morphology of the ZnO thin films can be controlled through the cooling process, and the morphology of ZnO thin films may change with cooling time and temperature. To verify the mechanism of the TDA method that we proposed, we annealed sol–gel spin-coated ZnO thin films under various conditions, and Figs. S3 and S4 show FE-SEM images of the ZnO thin films annealed by using the TDA method with various annealing conditions. When the sol–gel spin-coated ZnO thin films were annealed by using the TDA method with cooling time of 10 s, the size of the ZnO nanosheet was smaller than that of the ZnO nanosheet annealed by using the TDA method with a cooling time of 30 s, and the density of the ZnO nanosheet increased. In contrast, when the sol–gel spin-coated ZnO thin films were annealed by using the TDA method with a longer cooling time (180 s), the size of the ZnO nanosheet was larger than that of ZnO thin films annealed by using the TDA method with a shorter cooling time (10 s and 30 s), as shown in Fig. S3. The difference in the nanosheet size and density can be explained by the number of moving ZnO molecules during the annealing process. With respect to the short cooling time, the ZnO molecules at the surface and bottom of the ZnO wrinkled structures moved readily because of the relatively low temperature difference, which caused the easy formation of the ZnO nanosheets, an increase in the nanosheet density, and a decrease in the nanosheet size. In contrast, when the ZnO thin films experienced a long cooling time before the annealing process, the ZnO molecules at the surface of the wrinkled structures also readily moved to the bottom of the ZnO wrinkled structures due to the direct thermal energy. However, the movement of the ZnO molecules at the bottom of the wrinkled structures was more restrained than that of the ZnO molecules with a short cooling time. Consequently, the movement difference between the ZnO molecules at the surface and bottom of the wrinkled structures increased the size of the ZnO nanosheet; however, the density of the ZnO nanosheets decreased due to the restrained movement of the ZnO molecules at the bottom of the wrinkled structures. Furthermore, the number of graphene-like ZnO nanosheets decraesed with increasing temperature of the cold plate, and no ZnO nanosheets were observed in the ZnO thin films annealed with the TDA method with a cold plate temperature of 20 °C, as shown in Fig. S4a and b. This phenomenon occurred because the temperature increase of the cold plate caused a small temperature difference between the ZnO molecules at the surface and bottom of the wrinkled structures, decreasing the mobility of the ZnO molecules. Consequently, these results indicate that the formation of ZnO nanosheets with the TDA method depends on the temperature of the cold plate and cooling time before the annealing process. High-resolution transmission electron microscopy (HRTEM) was employed to study the morphology and structure of the ZnO thin films annealed with the TDA method. A HRTEM image of the ZnO thin films annealed through the TDA method shown in Fig. 5a reveals clear and continuous ordered lattice fringes and almost no disordered areas can be seen, indicating the single-crystalline nature of the ZnO thin films. The measured interplanar distance of 0.26 nm agrees well with the *d*-spacing of (002) planes of the hexagonal wurtzite ZnO^21^. Figure 5b shows the selected-area electron diffraction (SAED) pattern obtained from the HRTEM analysis. As shown in Fig. 5b, many regularly distributed reflections are observed. Analyzing the spot geometry and the associated lattice distances, the center spot (red circle) was attributed to ZnO thin films with a [100] zone axis orientation, and the others were due to the ZnO thin films. The HRTEM and SAED patterns indicated that the preferred growth direction of the single-crystal ZnO thin films annealed by the using TDA method was parallel to the (002) direction (*c*-axis).

**Figure 6:**
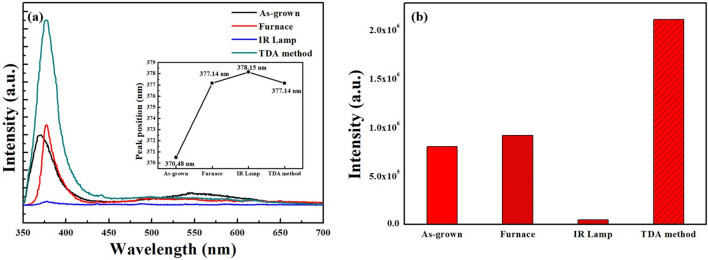
**(a)** PL spectra and **(b)** the intensity of NBE emission of sol–gel spin-coated ZnO thin films annealed by using various annealing method, and NBE emission peak position (inset).

Figure 6a shows the photoluminescence (PL) spectra of the ZnO thin films after various annealing methods and all PL spectra consisted of sharp and broad emissions in the UV and visible regions, respectively. In general, the sharp and strong emission observed in the UV region is referred to as a near-band-edge (NBE) emission and is attributed to the recombination of free excitons, whereas the weak and broad emission observed in the visible region is referred to as a deep-level (DL) emission and is ascribed to defects in the ZnO lattice, such as zinc vacancies, oxygen vacancies, interstitial zinc, and interstitial oxygen^22–26^. As shown in Fig. 6a,b, the NBE emission intensity of the ZnO thin films annealed by using TDA method clearly increased by a factor of two compared to that of the ZnO thin films annealed with a furnace in air. In particular, despite using the same thermal source (IR lamp), the ZnO thin films annealed with the TDA method exhibited an increased NBE emission intensity by a factor of 44 compared to that of the ZnO thin films annealed without a cooling process. There were two reasons for the increased NBE emission intensity. The first reason is that the ZnO thin films annealed with the TDA method were single crystal, as confirmed in the HRTEM and SAED patterns. This means that the crystallinity of the ZnO thin films improved and the number of defects that interrupt the recombination of free excitons also decreased. Second, the surface-to-volume ratio of the ZnO thin films annealed by using the TDA method increased owing to the smaller size of the ZnO particles than that of thin films annealed using the furnace and IR lamp, causing additional absorption and emission. Furthermore, as shown in the inset of Fig. 6a, the position of the NBE emission center of ZnO thin films redshifted after the annealing process, and these phenomena proved the crystallization of the ZnO thin films^27^.

### Performance of ZnO based UV photodetectors

**Figure 7:**
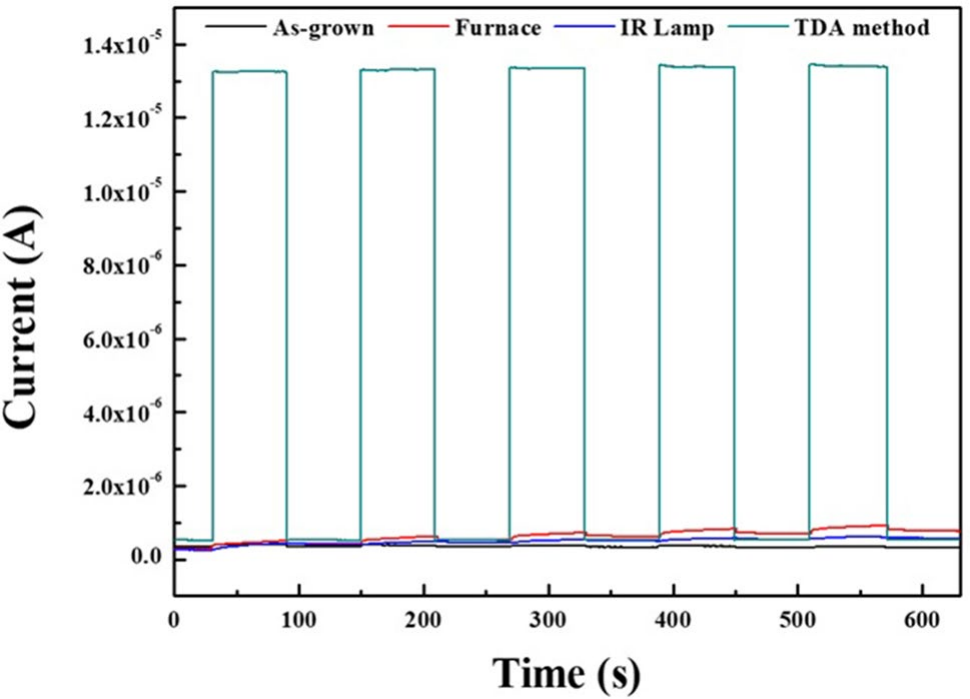
Time-dependent UV photoresponse of non-annealed ZnO thin films and annealed ZnO thin films by using furnace, IR lamp, and TDA method.

**Figure 8:**
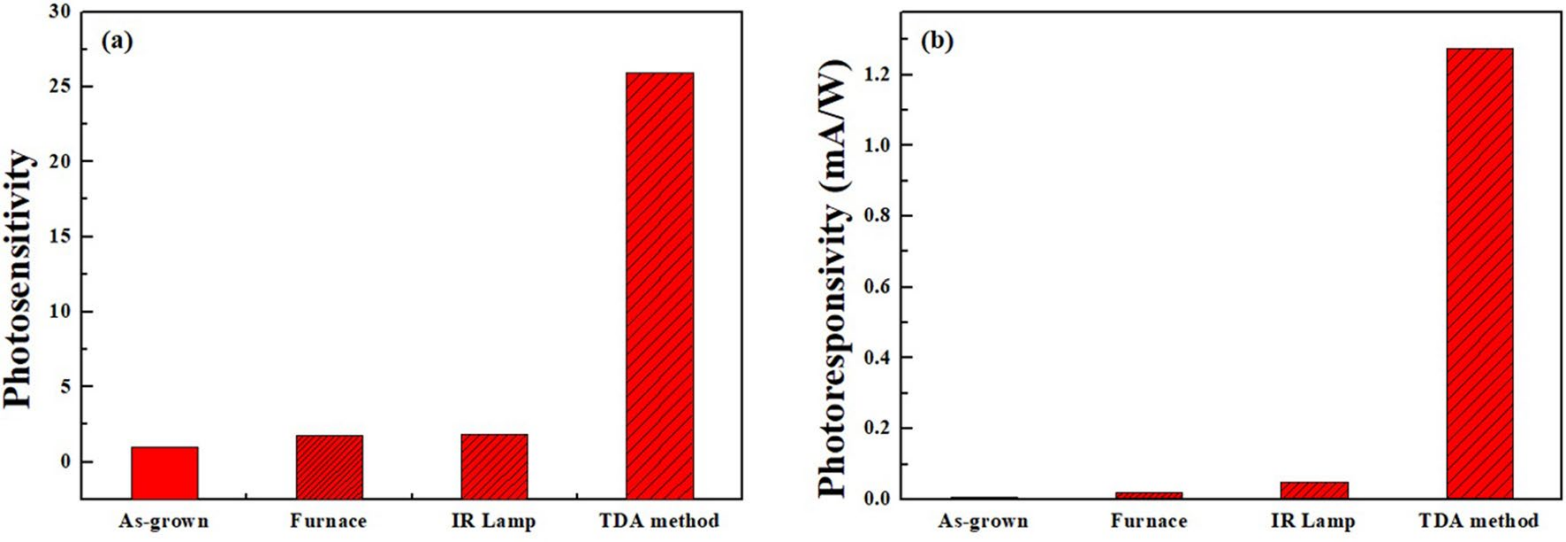
**(a)** Photosensitivity and **(b)** photoresponsivity of non-annealed ZnO thin films and annealed ZnO thin films by using furnace, IR lamp, and TDA method.

**Figure 9:**
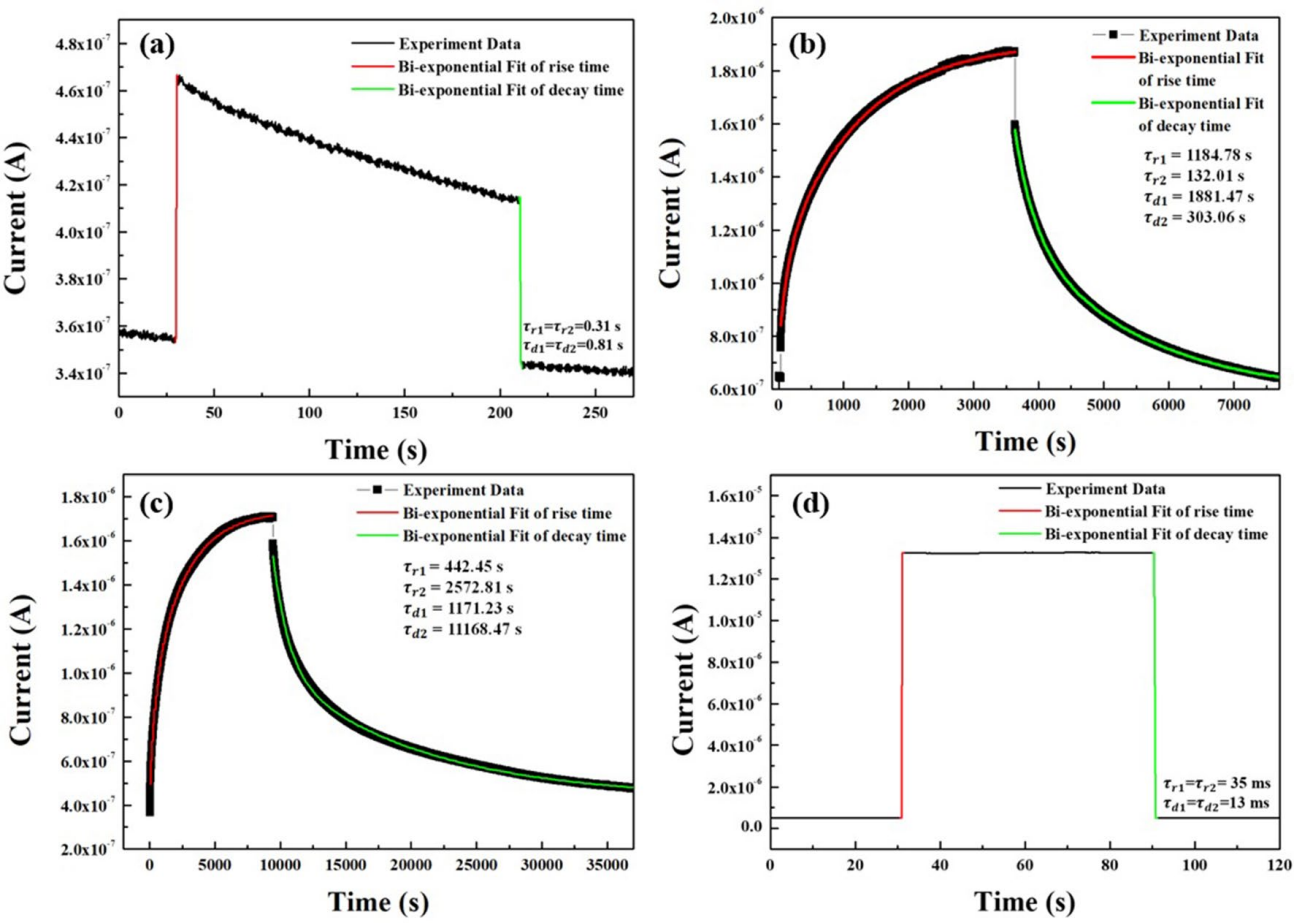
Rise and decay of photoresponse curves for **(a)** non-annealed ZnO thin films and annealed ZnO thin films by using **(b)** furnace, **(c)** IR lamp, and **(d)** TDA method.

To investigate the effect of the annealing methods on the UV photoresponse of the ZnO thin films, we fabricated metal–semiconductor–metal (MSM) UV photodetectors based on ZnO thin films. Thus, MSM UV photodetector was fabricated by depositing indium (In) electrode onto the ZnO thin films via thermal evaporation with metal shadow mask. Figure S5 illustrates the shematic diagram of MSM UV photodetector and the active area of UV photodetector was about 1.6 cm^2^. Figure 7 shows the time-dependent photoresponse of the ZnO thin films measured by periodically turning on and off the UV light (λ = 365 nm) in air. With respect to ZnO thin films annealed by using the TDA method, as soon as the UV light was turned on, the photocurrent rapidly increased and saturated immediately, showing excellent stability and reproducibility. In addition, the photocurrent value of the ZnO thin films annealed by the TDA method clearly increased by a factor of 30 compared to that of the other ZnO thin films under the same measurement conditions (power density of UV light, temperature, and humidity). In contrast, as-grown and annealed ZnO thin films prepared using the furnace and IR lamp exhibited extremely low photocurrent values. For an accurate comparison, the individual UV photoresponse properties of ZnO thin films are shown in Fig. S6. In the case of the unannealed ZnO thin films, the photocurrent saturated as soon as the UV light was turned on, and the current increment was very slight (51 nA). In addition, although the photocurrent response of the ZnO thin films annealed by using the furnace and IR lamp was improved compared to that of the unannealed ZnO thin films, the value of the dark current and the photocurrent of ZnO thin films annealed by using the furnace and IR lamp gradually increased during repetitive on and off cycles with the UV light, indicating the poor reproducibility of the photocurrent. The mechanism for the photocurrent rise and decay from the ZnO thin films is as follows. When the ZnO thin film-based UV photodetector is exposed to air in the dark, the oxygen molecules are adsorbed on the ZnO thin film surface and capture free electrons from the ZnO thin films, forming a depletion region near the surface and causing a decline in conductivity of the ZnO thin films (O_2_ + e^−^ → O_2_^−^)^28–30^. When the ZnO thin film based UV photodetector is exposed to UV light, electron–hole pairs generateed, and some photogenerated holes migrate to the surface of the ZnO thin films to combine with electrons that capture the oxygen molecules, resulting in a reduction in the depletion barrier thickness (h^+^  + O_2_^−^ → O_2_)^31,32^. At the same time, the photogenerated electrons move to the electrode based on the bias voltage, resulting in a photocurrent increase. When the UV light is turned off, the photogenerated electron–hole pairs instantly recombine with each other and the readsorption of the oxygen molcules occurs on the surface of the ZnO thin film, resulting in a decrease in the photocurrent^30^. Therefore, in the case of the non-annealed ZnO thin films, a gradual decrease in the photocurrent under UV illumination was observed because the amount of readsorbed oxygen molecules combined with free electrons was greater than that of the photogenerated electrons, and the cracks between the wrinkled network structures also interrupted the movement of free electrons, causing a decrease in the photocurrent. With respect to ZnO thin films annealed by using the furnace and IR lamp, the typical drawback of ZnO thin film-based UV photodetectors is the slow rise and decay of the photocurrent. When UV illumination starts, the amount of photogenerated electrons is greater than that of readsorbed oxygen molecules, but the readsorbed oxygen molecules combined with the free electrons increase with illumination time and cause a slow increase in the photocurrent. In addition, when the UV illumincation is turned off, an immediate recombination of photogenerated electron–hole pairs occurs, which results in rapid decrease in the photocurrent at the early part of the decay process, and readsorption of oxygen molecules that react with free excitons occurs on the surface of the ZnO thin films, which leads to a slow decrease in the photocurrent. However, ZnO thin films annealed by using the TDA method show a rapid increase and decrease in the photocurrent, and a stable photocurrent occurred during the repetitive on and off cycling of the UV light. This was due to a high surface-to-volume ratio that increased the amount of captured oxygen ions on the surface of the ZnO thin films and the perfect single crystal structure of the ZnO thin films. Furthermore, to compare the performance of the UV photodetectors, we calculated the photosensitivity and photoresponsivity of the ZnO thin films by using the Eqs. (1) and (2)^33–35^.

1 $$S=\frac{{I}_{ph}}{{I}_{dark}}$$

2 $$\mathrm{R}=\frac{{I}_{ph}-{I}_{dark}}{{P}_{op}}$$

where *S* is the photosensitivity of the ZnO thin films, *I*_*ph*_ is the photocurrent, *I*_*dark*_ is the dark current, *R* is the photoresponsivity, and *P*_*op*_ is the optical power of the UV source (10 mW/cm^2^). As shown in Fig. 8, the photosensitivity of the ZnO thin films annealed with the TDA method clearly increased by a factor of approximately 15 compared to that of the other samples. In particular, the photoresponsivity of ZnO thin films annealed by using the TDA method increased by a factor of 228, 60, and 26 compared with that of the unannealed ZnO thin films, ZnO thin films annealed in the furnace and ZnO thin films annealed with an IR lamp, respectively. This means that the TDA method is a suitable method for resolving the typical drawback of ZnO thin film-based UV photodetectors that have a low on/off current ratio, low sensitivity and poor photocurrent stability. Furthermore, to investigate the photoresponse speed of the ZnO thin films with various annealing methods, we calculated the rise and decay time constant of the photocurrents by using biexponetial fit. The following two equations were used for the rise and decay time constants of the photocurrent^36^:

3 $$I\left(t\right)={I}_{0}+{A}_{1}\left(1-{e}^{-\frac{t}{{\tau }_{r1}}}\right)+{A}_{2}(1-{e}^{-\frac{t}{{\tau }_{r2}}})$$

4 $$I\left(t\right)={I}_{0}+{A}_{3}{e}^{-\frac{t}{{\tau }_{d1}}}+{A}_{4}{e}^{-\frac{t}{{\tau }_{d2}}}$$

where *I*_*0*_ is the dark current ; *A*_*1*_, *A*_*2*_, *A*_*3*_, and *A*_*4*_ are positive constants ; *τ*_*r1*_ and *τ*_*r2*_ are the rise time constants ; and *τ*_*d1*_ and *τ*_*d2*_ are the decay time constants. Based on the curve fitting, the estimated rise (*τ*_*r1*_ and *τ*_*r2*_) and decay (*τ*_*d1*_ and *τ*_*d2*_) time constants of the unannealed ZnO thin films were 0.31 and 0.81 s, respectively. In the case of the ZnO thin films annealed in a furnace and with an IR lamp, the rise time constants (*τ*_*r1*_ and *τ*_*r2*_) were 1184.78 s and 132.01 s and also 442.45 s and 2572.81 s, respectively, and the decay time constants (*τ*_*d1*_ and *τ*_*d2*_) were 1881.47 s and 303.06 s and also 1171.23 s and 11,168.47 s, respectively. For the ZnO thin films annealed with the TDA method, the rise (*τ*_*r1*_ = *τ*_*r2*_) and decay (*τ*_*d1*_= *τ*_*d2*_) time constants were 35 ms and 73 ms, respectively. As shown in Fig. 9, the increase in the photocurrent under UV illumination consisted of a fast and slow process, and the fast rise process was attributed to the photogenerated electrons excited by the UV illumination, whereas the slow rise process was governed by readsorption of oxygen molecules on the ZnO surface. After turning off the UV light, the decay of the photocurrent also consisted of a fast and slow process, and the fast decay process was attributed to the recombination of photogenerated electron–hole pairs, whereas the slow decay process resulted from the readsorption of oxygen molecules on the ZnO surface. Although the non-annealed ZnO thin films had fast rise and decay time constants compared to those of the ZnO thin films annealed in the furnace and with an IR lamp, the increment in the photocurrent was very small, and the photocurrent decreased during exposure to the UV light. In contrast to the other annealing methods, the ZnO thin films annealed by using the TDA method clearly had faster rise and decay time constants with a great photocurrent stability, high on/off current ratio, and high sensitivity.

## Discussion and conclusion

In conclusion, we deposited the ZnO thin films onto Si substrates by using the sol–gel spin-coating method and annealed the ZnO thin films with the TDA method which eliminated heat from the substrates to form the mobility difference of ZnO molecules. The morphology of the ZnO thin films annealed by using the TDA method changed from wrinkled network structure to a graphene-like nanosheet and the crystal phase also changed from amorphous to that of a single crystal. The photocurrent value of the ZnO thin films annealed by the TDA method clearly increased by a factor of 30 compared to that of the other ZnO thin films. In addition, the ZnO thin films annealed by the TDA method showed improved UV photodetector performance compared to the ZnO thin films annealed in the furnace and by the IR lamp, such as excellent stability and reproducibility, fast rise and decay time constants, high on/off ratio, and high photosensitivity and photoresponsivity. This indicates that the TDA method is an effective route for the fabrication of high-performance UV photodetectors with a fast response speed, high photosensitivity and photoresponsivity, and great photocurrent stability.

## Experimental

ZnO thin films were deposited onto Si substrates by using the sol–gel spin-coating method and annealed in a furnace, with an IR lamp, and with the TDA method. A ZnO sol–gel solution was prepared by dissolving zinc acetate dihydrate and monoethanolamine (MEA) in 2-methoxyethanol to deposit ZnO thin films onto Si and PEN substrates. MEA was used to stabilize the solution and enhance the solubility of the precursor salt. The zinc acetate/MEA molar ratio equaled 1:1, and the concentration of the ZnO precursor solution was 0.5 M. The prepared mixture was stirred at 60 °C for 2 h to obtain a clear homogeneous solution that was used as a coating source after being cooled to room temperature. After preparing the sol–gel solution, the Si substrates were first cleaned in a piranha solution, which is a mixture of sulfuric acid (H_2_SO_4_) and hydrogen peroxide (H_2_O_2_), and then cleaned in hydrofluoric acid (HF). Then, the cleaned substrates were rinsed with deionized water for 2 min and dried with nitrogen (N_2_) gas (99.9999%). Then, the Si substrates were cleaned by ultrasonication in acetone and ethyl alcohol for 10 min, rinsed with distilled water for 2 min, and dried with nitrogen gas (99.9999%). The prepared sol–gel solution was deposited onto the cleaned Si substrates and spin-coated at 2000 rpm for 20 s and the spin-coated ZnO thin films were placed in the oven at 150 °C for 10 min for pre-heat treatment. After a deposition and pre-heat annealing process, the spin-coating and preheating processes was repeated nine additional times. Finally, the ZnO thin films obtained by repeating spin-coating and preheating processes with 10 times were post-heated at 500 °C for 1 h in the furnace, with the IR lamp, and with theTDA method to crystallize the amorphous state ZnO thin films. With respect to the TDA method, before the annealing process, the temperature of the ZnO thin films was decreased to − 10 °C for 30 s by a cooling plate. After 30 s, the ZnO thin film was annealed at 500 °C for 1 h using an IR lamp while maintaining the cooling process. The morphology of the ZnO thin films was measured by FE-SEM (TESCAN NIRA3LM) on an instrument with an accelerating voltage of 30 kV and FE-TEM (FEI TF30ST) on an instrument with a voltage of 300 kV and SAED capability. The crystal phases were analyzed by XRD (PANalytical X`Pert Pro) using a Cu-Kα radiation source (λ = 0.15406 nm) at an accelerating voltage of 40 kV. The optical properties were analyzed with PL measurements performed using a He-Cd laser with an excitation power of 20 mW and equipped with a 0.75 m single-grating monochromator with a photomultiplier tube. In addition, the photoresponse changes were measured at a bias voltage of 0.1 V using a UV light (λ = 365 nm) with a power density of 10 mW/cm^2^.

## Supplementary Information

Supplementary Figures.
