# Supplementary material for: Crystallization of ZnO thin films via thermal dissipation annealing method for high-performance UV photodetector with ultrahigh response speed
Source: Sci Rep. 2021 Jan 11;11:382. doi: 10.1038/s41598-020-79849-z (PMC7801514; doi:10.1038/s41598-020-79849-z)
Supplement: Supplementary file 1 — Supplementary Figures. [file 41598_2020_79849_MOESM1_ESM.pdf]

Supplementary material

Crystallization of ZnO thin films via thermal dissipation annealing method for high-performance UV photodetector with ultrahigh response speed

Dongwan Kim[1] and Jae-Young Leem[1,*]

[1]Department of Nanoscience & Engineering, Inje University, 197, Inje-ro, Gimhae-si, Gyeongsangnam-do 621-749, Republic of Korea

*Corresponding authors.

E-mail addresses: jyleem@inje.ac.kr

Tel.: +82-55-320-3716. Fax: +82-55-320-3631

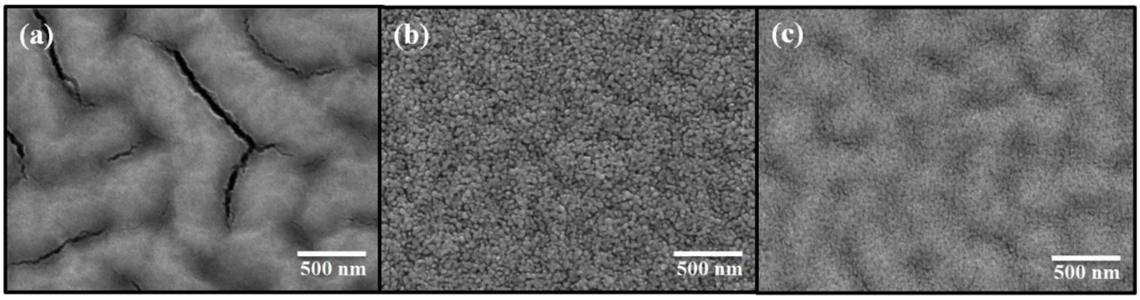

Figure S1. FE-SEM images of sol-gel spin-coated ZnO thin films annealed by using various method. (a) non-annealed ZnO thin films, (b) furnace, and (c) IR lamp.

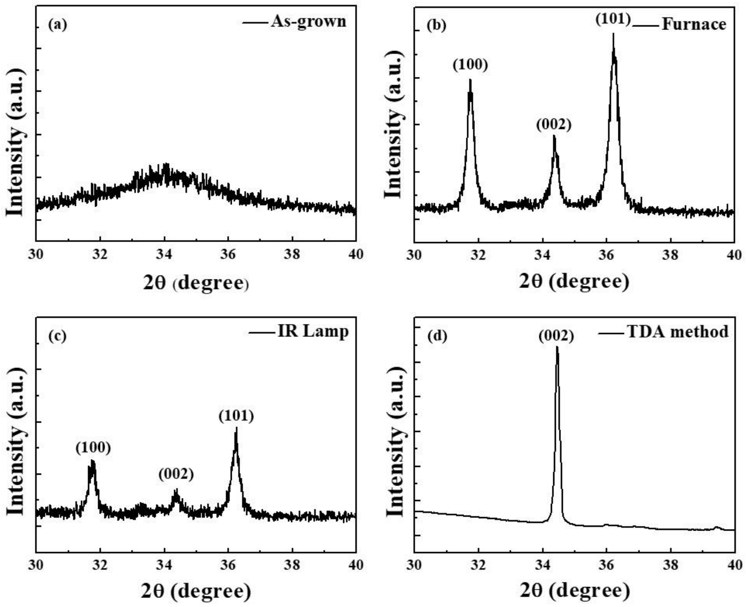

Figure. S2. X-ray diffraction patterns of sol-gel spin-coated ZnO thin films annealed by using various method. (a) non-annealed ZnO thin films, (b) furnace, (c) IR lamp, and (d) TDA method.

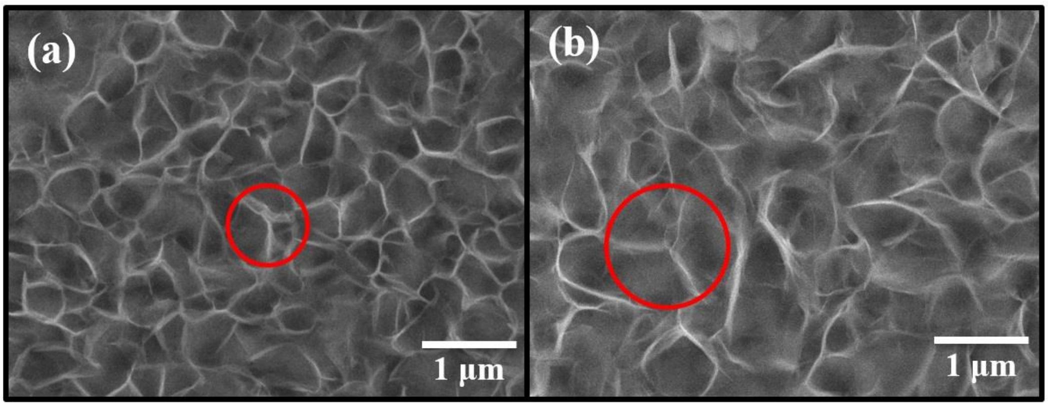

Figure S3. FE-SEM images of sol-gel spin-coated ZnO thin films annealed by using TDA method with cooling time change of (a) 10 and (b) 180 s, respectively.

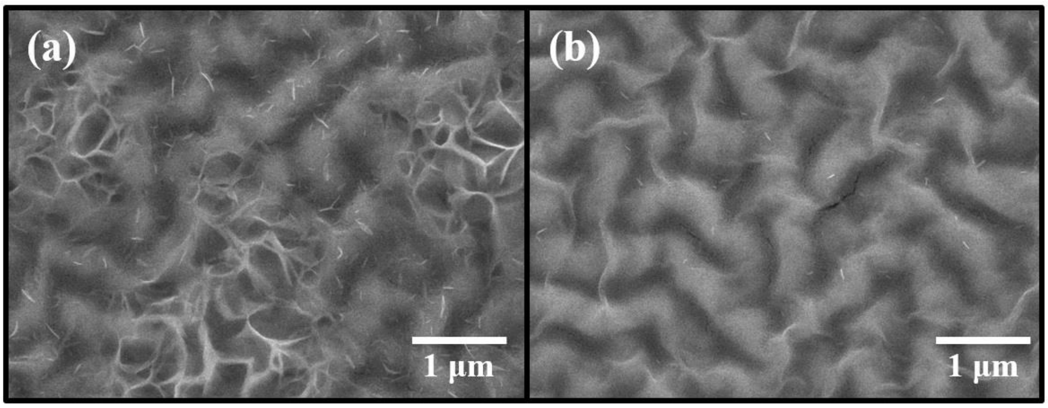

Figure S4. FE-SEM images of sol-gel spin-coated ZnO thin films annealed by using TDA method with cold plate temperature of (a) 10 and (b) 20 °C, respectively.

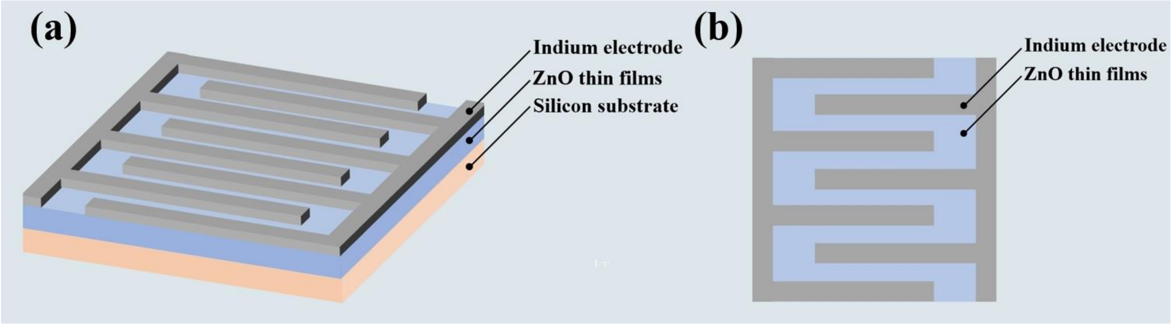

Figure S5. (a) Schematic diagram of the MSM UV photodetector and (b) top-view of MSM UV photodetector (figures not drawn to scale).

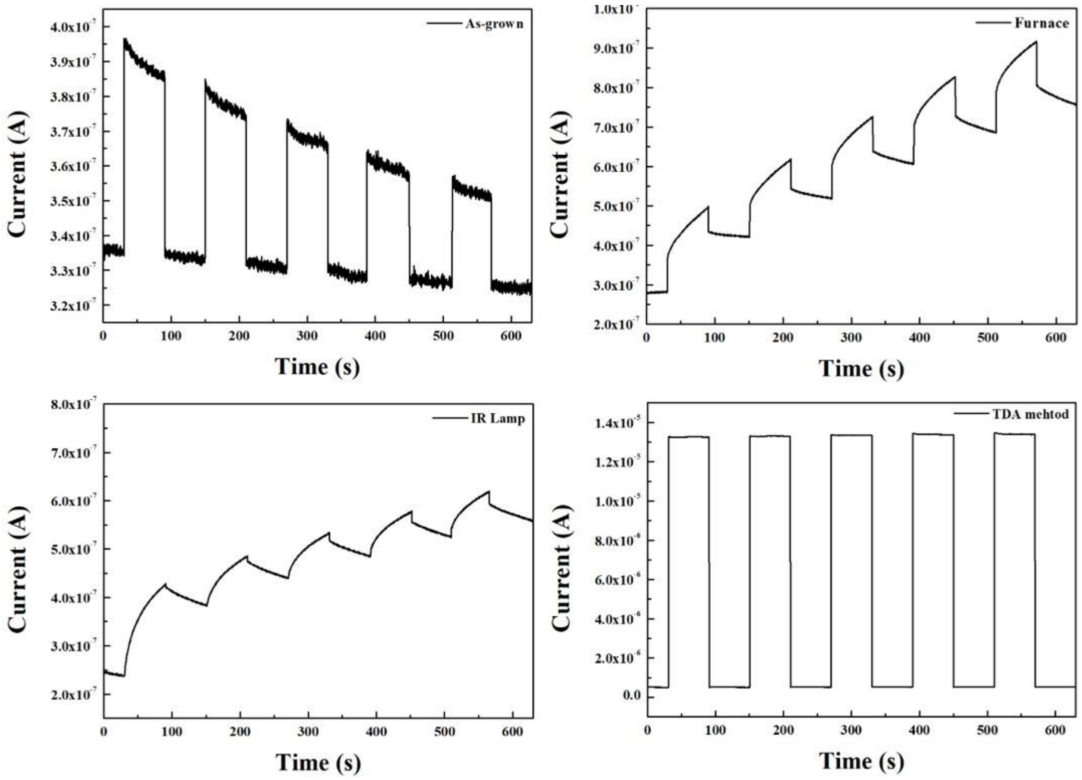

Figure S6. Time-dependent UV photoresponse of (a) non-annealed ZnO thin films and annealed ZnO thin films by using (b) furnace, (c) IR lamp, and (d) TDA method.
